# Development of an external system for monitoring the couch speed in radiotherapy using continuous bed movement

**DOI:** 10.1002/acm2.14497

**Published:** 2024-09-12

**Authors:** Hidetoshi Shimizu, Osamu Nakamura, Koji Sasaki, Takahiro Aoyama, Tomoki Kitagawa, Takeshi Kodaira

**Affiliations:** ^1^ Department of Radiation Oncology Aichi Cancer Center Hospital Nagoya Aichi Japan; ^2^ QUALITA Co., Ltd. Matsumoto Nagano Japan; ^3^ Graduate School of Radiological Technology Gunma Prefectural College of Health Sciences Maebashi Gunma Japan

**Keywords:** couch speed, quality assurance, Radixact, third party, tomotherapy

## Abstract

**Purpose:**

Total body irradiation before bone marrow transplantation for hematological malignancies using Radixact, a high‐precision radiotherapy machine, can potentially reduce side effects and the risk of secondary malignancies. However, stable control of couch speed is critical, and direct assessment methods outlined in quality assurance guidelines are lacking. This study aims to develop a real‐time couch speed verification system for the Radixact.

**Methods:**

The developed system used a linear encoder to measure couch speed directly. Accuracy was verified via a linear stage, comparing measurements with a laser distance sensor. After placing a phantom simulating the human body on the Radixact couch, the couch speed was verified using predefined speed plans.

**Results:**

Operating the linear stage at 0.1, 0.5, and 1.0 mm/s revealed that the maximum position error of the developed verification system compared to the laser distance sensor was nearly equivalent to the distance resolution of the system (0.05 mm/pulse), with negligible average speed error. When the Radixact couch operated at 0.1, 0.5, and 1.0 mm/s, the values obtained by the verification system agreed with the theoretical values within the sampling period (0.01 s) and distance resolution (0.05 mm).

**Conclusion:**

The verification system developed provides real‐time monitoring of the speed of the Radixact table, ensuring treatment effectiveness and patient safety. It would guarantee the couch speed's soundness and contribute to the “visualization” of safety.

## 1 INTRODUCTION

In hematological malignancies such as acute leukemia, whole‐body irradiation (total body irradiation, TBI) is performed before bone marrow transplantation to suppress the immune response.^1^ A major issue with TBI in children is the relatively higher radiosensitivity of normal tissues in them than in adults.^2^ It may be associated with adverse effects on neurocognitive, growth, endocrine, and metabolic functions after treatment.^3^ Additionally, the risk of secondary carcinogenesis should also be considered due to the expected long‐term prognosis.^3^, ^4^

Radixact is a high‐precision radiotherapy machine that uses a helical delivery technique in which the couch continuously moves while the gantry rotates during treatment, allowing the radiation to be focused on the tumor and reducing the dose to normal tissue. Therefore, compared with conventional 3D conformal radiotherapy, the radiation dose is uniform throughout the body, and the effects on normal tissues such as those of the lungs, kidneys, and lenses are minimized.^5^, ^6^ The use of Radixact is expected to reduce the side effects and the risk of secondary carcinogenesis; however, stable operation of the radiotherapy machine is a prerequisite. The stability of the couch speed during treatment is especially ensured only by monitoring and interlocks in the system, and the user cannot check for themselves whether the couch speed is appropriate. The continuous control of the couch speed is important during TBI, where the irradiated length is more and the irradiation time is much longer than in other treatment areas because Radixact employs a helical delivery technique.

The quality assurance guidelines for Radixact include the American Association of Physicists in Medicine task group (AAPM TG) 148 and 306, which do not include a method of directly assessing the couch speed. However, couch speed verification is recommended in these guidelines.^7^, ^8^ In addition, to date, no reports have evaluated the reliability of Radixact's couch speed using third‐party tools. Therefore, we developed a couch speed verification system to measure and monitor whether the couch speed operates properly in real time. We also investigated the possibility of measuring the couch speed of Radixact.

## 2 METHODS AND MATERIALS

### 2.1 Couch speed verification system

**FIGURE 1:**
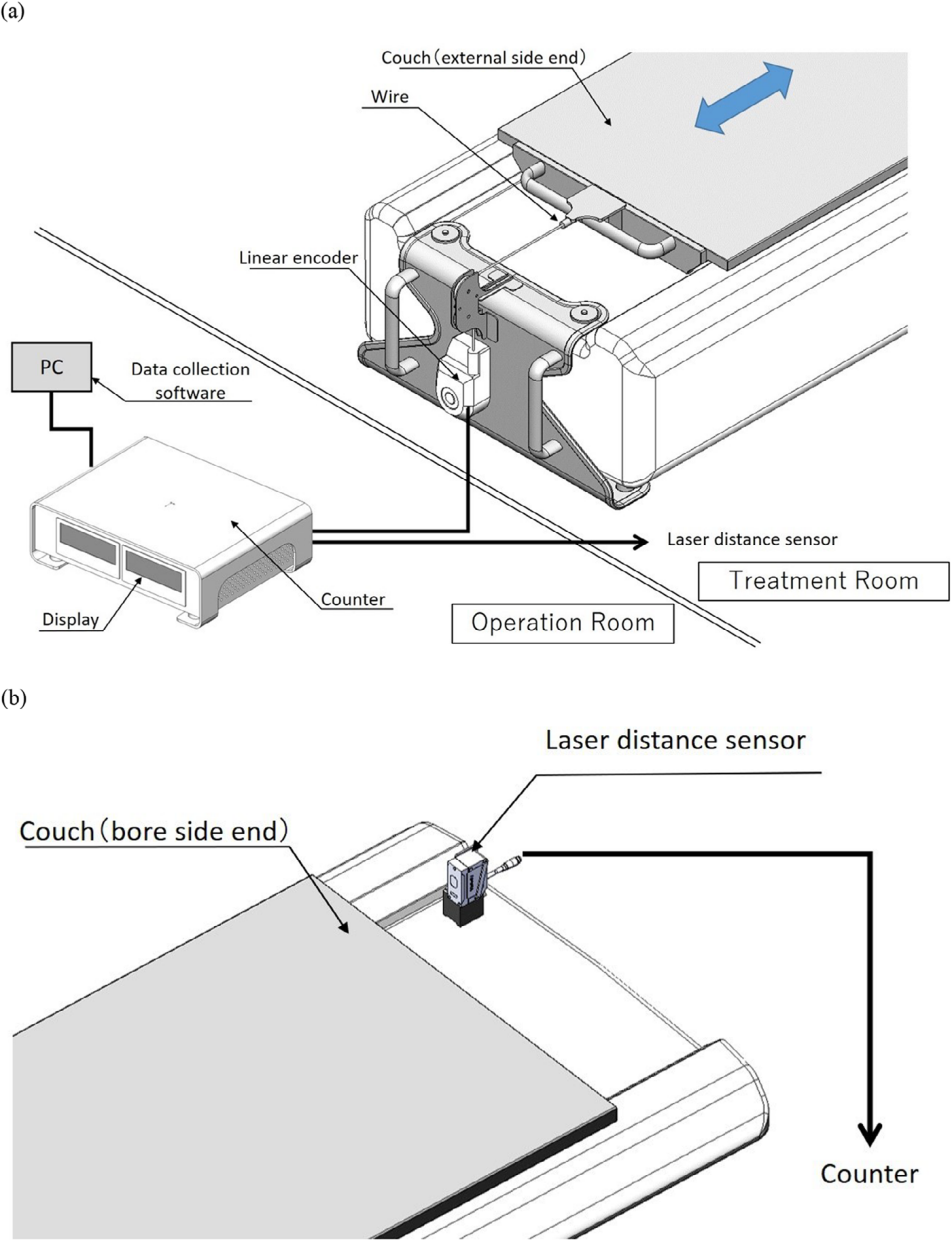
The configuration of the developed couch speed verification system (a). Pulse signals were acquired by connecting a wire attached to the external side of the Radixact couch to the rotary unit of the encoder. The pulse signals were output as quadruplicated by a data output counter, and the couch speed (cm/sec) and relative position (cm) were monitored via periodic calculations. The pulse signals were also acquired using data collection software to enable numerical analyses. A laser distance sensor was fixed at the bore side end of the couch to detect the starting position of the couch movement (b). DL, data logger.

In this study, we developed a system to measure the couch speed directly using a length‐measuring linear encoder. The configuration of the couch speed verification system is presented in Figure 1. A length‐measuring linear encoder (MUTOH, D‐1000Z‐E, distance resolution: 0.2 mm/pulse) was used in the verification system. By connecting a wire attached to the external side of the Radixact couch to the rotary unit of the encoder, pulse signals (relative position of the couch per 0.01 s sampling period) were acquired (Figure 1a). The pulse signals were output as quadruplicated (distance resolution: 0.05 mm/pulse) by a data output counter (CNT‐3204IN‐USB, CONTEC), and the couch speed (cm/s) and relative position (cm) were monitored by periodic calculation (MR45A1‐A‐EG, Henix). Table 1 shows the theoretical values of the variation range of the monitor display corresponding to the couch speed when the display period was changed to 0.1, 0.5, and 1.0 s. Although a large display period reduces the variation range, the sensitivity to changes in velocity (time resolution) is reduced because changes in the couch speed within the display period cannot be detected. In this study, a display period of 0.5 s was used, which is easily detected by humans when the couch moves in the actual clinical plan. The pulse signals were also acquired using data collection software to enable numerical analyses (Figure 1a). Since it is difficult to receive couch control pulses from the Radixact system due to system limitations, a laser distance sensor (Ono Sokki, LV‐8600, resolution: 0.07910 µm) was introduced, and the couch position detected by this sensor was used as the starting point of the couch movement. The laser distance sensor was fixed at the bore side end of the couch, as shown in Figure 1b, where it did not interfere with couch movement. The threshold of the laser distance sensor was set to 0.005 mm, a value sufficiently smaller than the distance resolution of the linear encoder.

**TABLE 1:** Variability in the monitor output in response to the combination with the couch speed and display period.

	Display period (s)		
Couch speed (mm/s)	1.0	0.5 (this study)	0.1
1.0	1.00–1.00	0.80–1.20	0.00–2.00
0.5	0.40–0.60	0.40–0.80	0.00–2.00
0.1	0.00–0.20	0.00–0.40	0.00–2.00

### 2.2 Accuracy verification of the couch speed verification system

**FIGURE 2:**
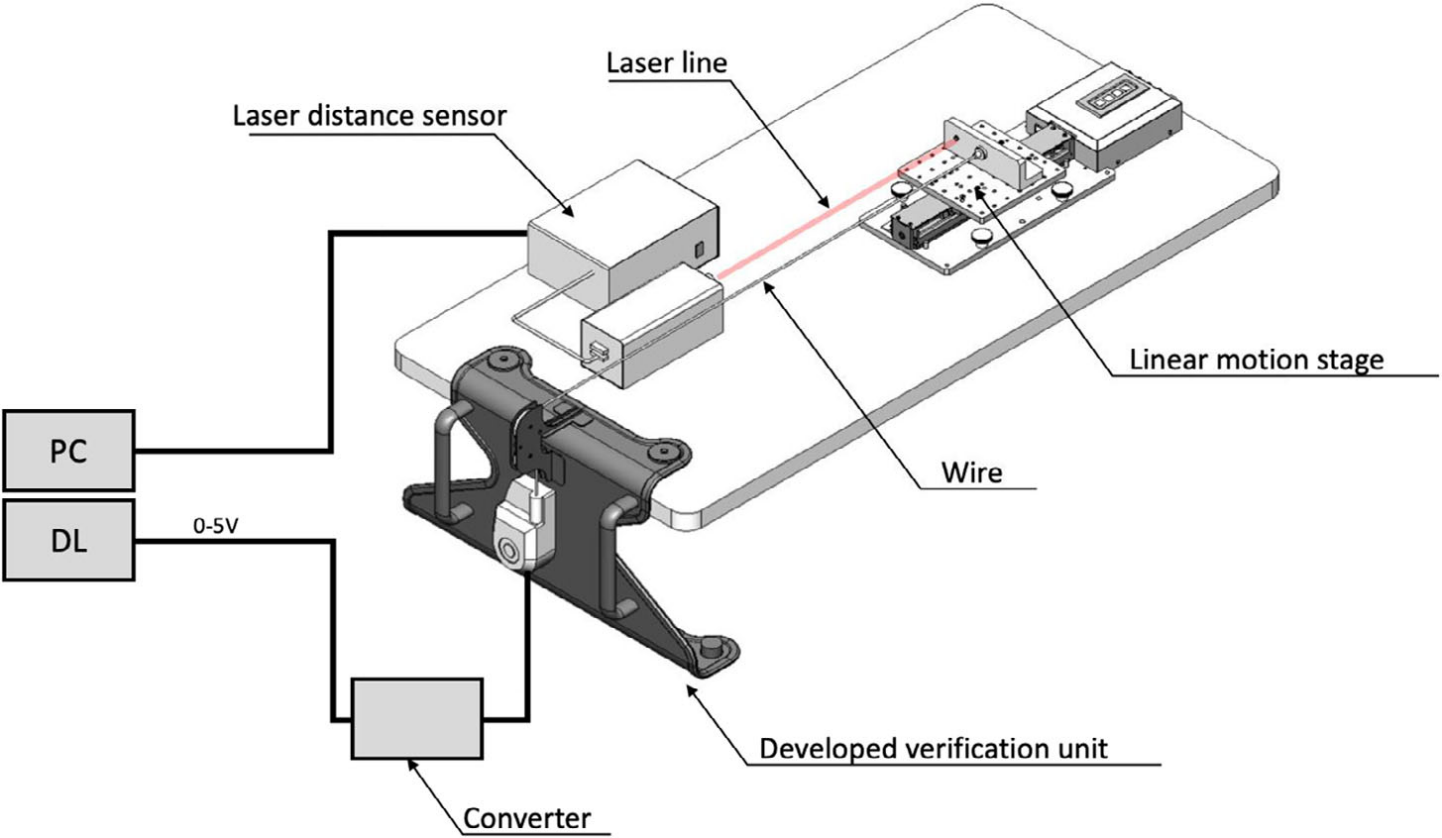
An arrangement for verifying the accuracy of the developed couch speed verification system. The linear motion stage was operated at speeds of 0.1, 0.5, and 1.0 mm/s for 60 s, and the stage positions were measured by a reference laser distance sensor and the developed verification system.

Figure 2 shows a configuration for verifying the accuracy of the developed couch speed verification system. The linear motion stage was operated at speeds of 0.1, 0.5, and 1.0 mm/s for 60 s, and the stage positions measured by the verification system were recorded in a data logger every 0.01 s sampling cycle.

At the same time, the position of the linear motion stage was recorded by a laser distance sensor (Ono Sokki, LV‐8600, resolution: 0.07910 µm), and the position detection accuracy of the verification system was verified based on this record. In addition, the average speed of the stage at 0.5 s, which was used as the display period of the monitor of the verification system, was calculated to compare the calculation accuracy of the average speed of the verification system with that of the laser distance sensor.

### 2.3 Verification of the couch speed of Radixact

Plans for verifying the known couch speeds (0.1, 0.5, and 1.0 mm/s) were created in the Machine Quality Assurance (QA) mode of the Radixact system. Each plan was set at 60 s. This study only evaluates the couch position accuracy; other planning parameters, such as the radiation field, are irrelevant. Thereafter, a phantom simulating the human body was set on the Radixact couch, in the arrangement shown in Figure 1. As shown in Figure 1a, the couch speed (cm/s) and relative position (cm) were monitored, and the pulse signals were recorded using data collection software to enable numerical analyses.

**FIGURE 3:**
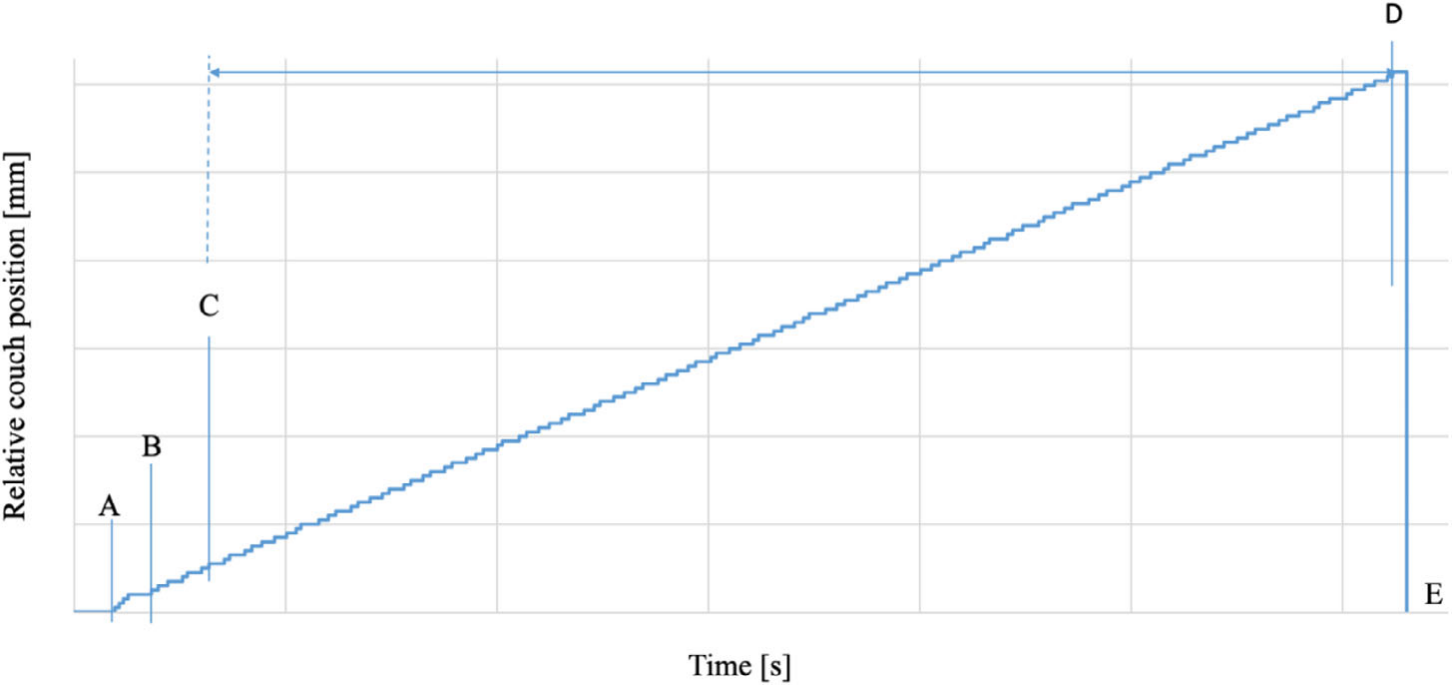
The relative position of the Radixact couch to the accumulated time when the plan was executed. When the irradiation button is pressed in the operation room, the Radixact couch starts moving at an unequal speed (A), then moves at a constant speed for stable operation (B), starts irradiation 2 s after moving (C), and after the plan is completed (D), returns to the designated position (E).

Figure 3 shows the relative position of the Radixact couch to the accumulated time when the plan was executed. When the irradiation button is pressed in the operation room, the Radixact couch starts moving at an unequal speed (A), then moves at a constant speed for stable operation (B), starts irradiation 2 s after moving (C), and after the plan is completed (D), returns to the designated position (E). At the same time, the pulse signal of the couch movement was detected by the laser distance sensor (Figure 1b), and the couch speed was analyzed for 60 s, from 2 to 62 s.

## 3 RESULTS

### 3.1 Accuracy verification of the couch speed verification system

When the linear motion stage was operated at 0.1, 0.5, and 1.0 mm/s for 60 s (sampling period: 0.01 s), the maximum position error of the developed couch speed verification system relative to the laser distance sensor was 0.065 , 0.067 , and 0.066 mm, respectively. These values were close to the distance resolution of the verification system (0.05 mm/pulse). Table 2 shows the average speed during 0.5 s at 0.1, 0.5, and 1.0 mm/s for 60 s of the linear motion stage. At the linear motion stage speed of 1.0 mm/s, the average speeds measured by the laser distance sensor and the verification system were 1.000 ± 0.056  and 0.980 ± 0.046 mm/s, respectively. The average speed error of the verification system was 0.020 mm/s. Similarly, the average speed error of 0.1 and 0.5 mm/s for 60 s of the linear motion stage was 0.002  and 0.010 mm/s, respectively.

**TABLE 2:** The velocity obtained from the laser distance sensor and the developed verification system (mm/s).

Velocity	0.1 mm/s		0.5 mm/s		1.0 mm/s	
System	Laser distance sensor	Our developed verification system	Laser distance sensor	Our developed verification system	Laser distance sensor	Our developed verification system
Average	0.100	0.098	0.500	0.490	1.000	0.980
S.D.	0.009	0.046	0.006	0.046	0.056	0.046
Minimum‐Maximum	0.071–0.135	0.000–0.200	0.474–0.530	0.400–0.600	0.973–1.029	0.900–1.100

### 3.2 Verification of the couch speed of Radixact

**FIGURE 4:**
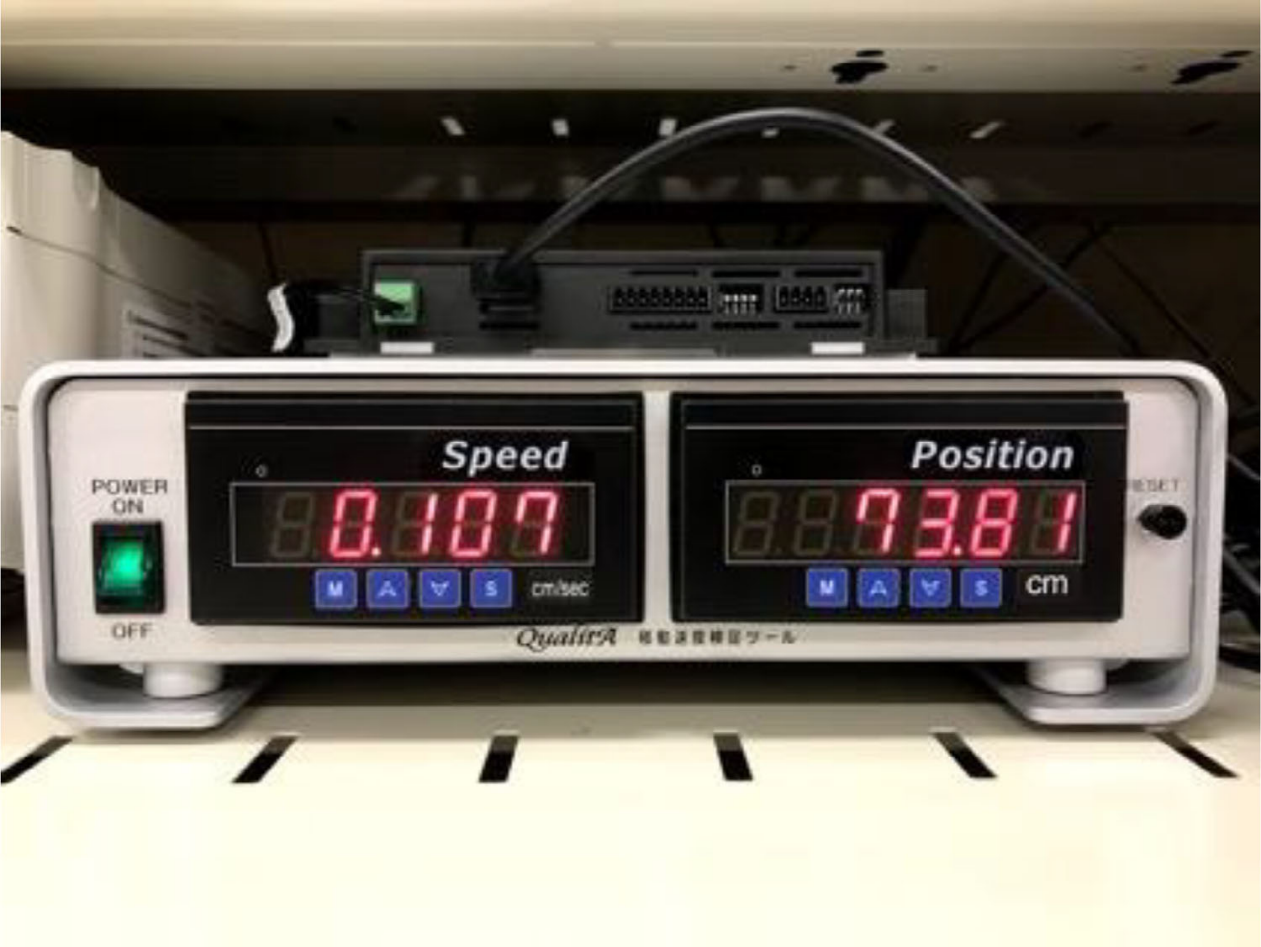
Monitor display when a plan with a couch speed of 1 mm/s is executed.

Figure 4 shows a photo of the monitor display when the plan of the couch speed of 1 mm/s was run. The display shows the couch speed (cm/sec) and relative position (cm) calculated by periodic calculation of the pulse signal from the length‐measuring linear encoder. The theoretical value of the variation range of the monitor display was 0.80–1.20 mm/s, as shown in Table 1 because the display period was 0.5 s. The monitor display values varied within the range of 0.87–1.15 mm/s for the couch speed of 1 mm/s (Supplementary file), which was within the theoretical value of the variation range. The monitor display values of the remaining couch speeds (0.1 and 0.5 mm/s) also varied within the theoretical values shown in Table 1.

**FIGURE 5:**
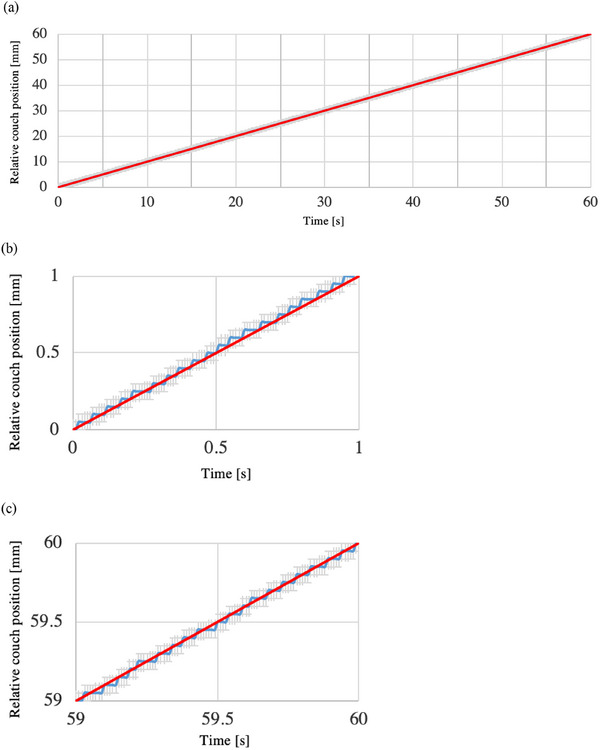
The relative couch position of the verification system to the sampling time at the couch speed of 1 mm/s [(a) overall, (b) 1 s after the start of the irradiation, and (c) 1 s before the end of the irradiation]. The blue lines show the measured values obtained by the verification system, while the red lines show the theoretical values (average couch speed of 1 mm/s for 60 s). The gray region shows the error bars resulting from adding the sampling period of 0.01 s on the horizontal axis and the distance resolution of 0.05 mm on the vertical axis to the verification system measurement.

**FIGURE 6:**
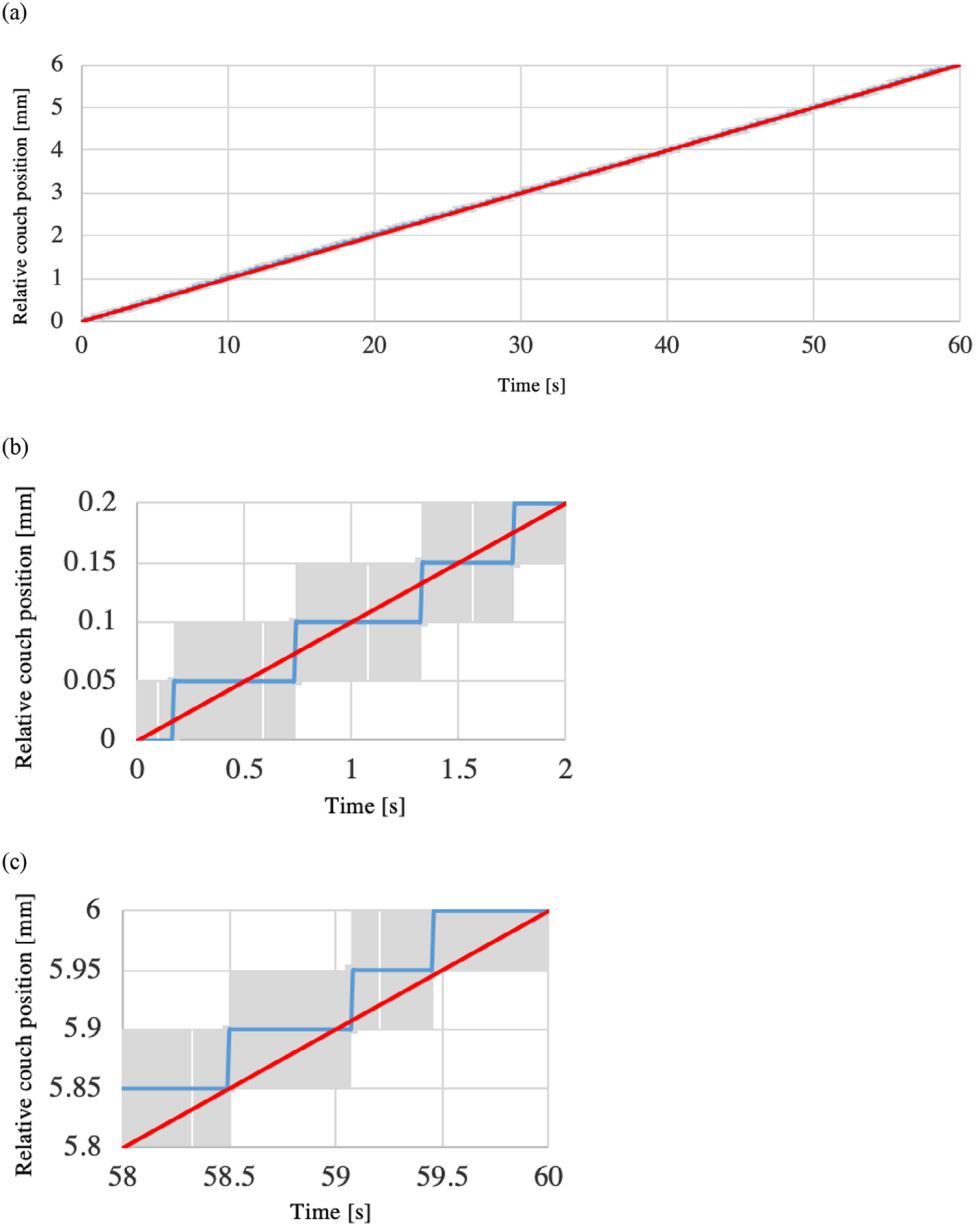
The relative couch position of the verification system to the sampling time at the couch speed of 0.1 mm/s [(a) overall, (b) 2 s after the start of the irradiation, and (c) 2 s before the end of the irradiation]. The blue lines show the measured values obtained by the verification system, while the red lines show the theoretical values (average couch speed of 1 mm/s for 60 s). The gray region shows the error bars resulting from adding the sampling period of 0.01 s on the horizontal axis and the distance resolution of 0.05 mm on the vertical axis to the verification system measurement.

Figure 5 shows the relative couch position of the verification system to the sampling time at the couch speed of 1 mm/s [(a) overall, (b) 1 s after the start of the irradiation, and (c) 1 s before the end of the irradiation]. The blue lines show the measured values obtained by the verification system, and the red lines show the theoretical values (average couch speed of 1 mm/s for 60 s). The gray region shows the error bars resulting from adding the sampling period of 0.01 s on the horizontal axis and the distance resolution of 0.05 mm on the vertical axis to the verification system measurement. The theoretical values were within the error bars of the values obtained by the verification system. Figure 6 shows the relative couch position of the verification system to the sampling time at the couch speed of 0.1 mm/s [(a) overall, (b) 2 s after the start of the irradiation, and (c) 2 s before the end of the irradiation]. The theoretical values were within the error bars of the values obtained by the verification system, as was the case for the couch speed of 1 mm/s (same for the couch speed of 0.05 mm/s).

## 4 DISCUSSION

We developed a couch speed verification system that can measure and monitor whether the couch speed is operating properly in real‐time and investigated the possibility of measuring the couch speed of the Radixact. As a basic study, when the linear motion stage was operated at 0.1, 0.5, and 1.0 mm/s, the maximum position error of the developed couch speed verification system relative to the laser distance sensor was almost the same as the distance resolution of the verification system (0.05 mm/pulse), and the average speed error was small. When the Radixact couch was operated at 0.1, 0.5, and 1.0 mm/s, the values obtained by the verification system agreed with the theoretical values within the sampling period (0.01 s) and distance resolution (0.05 mm).

In this study, the couch speed and relative position were displayed on a monitor by periodic calculation of the pulse signal of the developed couch speed verification system. When the Radixact couch was operated at 0.1, 0.5, and 1.0 mm/s, the variation range of the monitor display values was within the theoretical values shown in Table 1. Although the general couch speed in the TBI for children was not indicated, the average couch speed (min‐max) calculated from the couch travel distance and delivery time for the TBI reported by Gruen et al. was 1.0 mm/s (0.9–1.4 mm/s).^9^ When the couch speed is 1.0 mm/s, as shown in Table 1, it is possible to detect the speed variation of ± 20% or more of the couch speed at a display period of 0.5 s. A higher performance encoder is required to reduce the display cycle and variation range of the monitor and to evaluate the couch speed more accurately. For example, using a length‐measuring linear encoder with a distance resolution of 0.005 mm, it is possible to detect the speed variation of at least ± 2% as required by AAPM TG 148^7^ at a couch speed of 1.0 mm/s with the same display period of 0.5 s as in this study. In addition, the couch speed in treatment cases other than the TBI is generally around 0.5 mm/s.^10^ Table 1 shows that the variation range becomes larger at lower couch speeds; therefore, a higher performance encoder should be used if the verification system is used for other treatment cases.

Next, in the analysis of the pulse signals from the developed verification system, when the Radixact couch was operated at 0.1, 0.5, and 1.0 mm/s, the values obtained by the verification system agreed with the theoretical values within the sampling period (0.01 s) and distance resolution (0.05 mm); thus, it was confirmed that the Radixact couch was well controlled. The consistency of Radixact's couch speed is one of the QA items in AAPM 148, 306,^7^, ^8^ and there are two QA methods: a traditional method using film^7^ and the latest method using the Tomo Quality Assurance (TQA) software.^11^ In the film‐based method, the MLC is fully opened, and the couch is moved at a constant speed while being irradiated; a scanner reads the film, and a profile along the axis of couch movement is created from the results. The relative optical density on the profile is confirmed to be less than 2%; however, the result is affected by the variation in radiation output. On the other hand, in the TQA‐based method, like the film method, radiation is irradiated onto a staircase phantom, and the passed data is used to verify the reproducibility of the physical length of the stairs (without directly evaluating the couch speed). To the best of our knowledge, no previous reports have directly evaluated couch speed with the Radixact, and our proposed method of using the length‐measuring linear encoder is completely new.

We need to describe the difference between the Radixact delivery modes. The Radixact has two modes: TomoHelical mode, where the gantry rotates, and the TomoDirect mode, where the gantry is fixed. The above report^10^ was the general couch speed in the TomoHelical mode, and the couch speed in the TomoDirect mode (generally 1–8 mm/s) is outside the range verified in this study. Therefore, the results of this study cannot be directly extrapolated to the TomoDirect mode. However, as shown in Table 1, the theoretical value of the variation range is smaller and the detection accuracy is better because the encoder system generally increases the number of pulses per unit time as the couch speed increases. This system might well be applicable to the TomoDirect mode; however, additional verification would be required before use.

The couch speed verification system developed in this study can be applied not only to the Radixact but also to TBI using a treatment couch movement method other than the Radixact,^12^ PET/CT systems using a continuous couch movement method with variable speed,^13^ and diagnostic x‐ray CT systems. By objectively evaluating the speed of these products using a third‐party tool such as the length‐measuring linear encoder, we can ensure the safe use of the products and provide users with a sense of security by “visualizing” the safety of the products.

Nevertheless, this study has some limitations. First, while the study successfully developed a couch speed verification system and demonstrated its accuracy, there was no validation in clinical scenarios such as the patient plan. Second, the study focused solely on verifying the couch speed of the Radixact using a developed verification system. However, it did not evaluate the potential impact on treatment outcomes, such as improved treatment efficacy or reduced patient side effects. Finally, although the Radixact couch speed could be evaluated with the developed verification system, it is necessary to use a high‐performance linear encoder to evaluate the couch speed with even greater accuracy.

## 5 CONCLUSION

To evaluate the proper couch speed of the Radixact, which is expected to reduce the side effects of TBI for childhood and the risk of secondary malignancies, we developed a couch speed verification system that can be monitored and confirm the couch speed accuracy. This verification system can be easily attached to the external side of the Radixact couch without modifying the Radixact system. It would guarantee the soundness of the couch speed and contribute to the “visualization” of safety.

## AUTHOR CONTRIBUTIONS

Conceived the study, analyzed, and interpreted the data and drafted the manuscript: Hidetoshi Shimizu and Osamu Nakamura. Involved in the study design and significantly contributed to the editing of the manuscript: Koji Sasaki, Takahiro Aoyama, Tomoki Kitagawa, and Takeshi Kodaira. All authors read and approved the final manuscript.

## CONFLICT OF INTEREST STATEMENT

The authors declare no conflicts of interest.

## Supporting information

Supporting Information

## Data Availability

Raw data were generated at Aichi Cancer Center Hospital. Derived data supporting the findings of this study are available from the corresponding author (Hidetoshi Shimizu) on reasonable request.
